# Incidence and prevalence of idiopathic inflammatory myopathies among commercially insured, Medicare supplemental insured, and Medicaid enrolled populations: an administrative claims analysis

**DOI:** 10.1186/1471-2474-13-103

**Published:** 2012-06-15

**Authors:** Karen E Smoyer-Tomic, Anthony A Amato, Ancilla W Fernandes

**Affiliations:** 1 Thomson Reuters, 4301 Connecticut Ave. NW, Suite 330, Washington, DC 20008, USA; 2 Division of Neuromuscular Disease, Department of Neurology, Brigham and Women’s Hospital, Harvard Medical School, Washington, DC, USA; 3 Health Outcomes and Pharmacoeconomics, MedImmune, LLC., Gaithersburg, MD, USA

## Abstract

**Background:**

Idiopathic inflammatory myopathies (IIMs) are a rare group of autoimmune syndromes characterized by chronic muscle inflammation and muscle weakness with no known cause. Little is known about their incidence and prevalence. This study reports the incidence and prevalence of IIMs among commercially insured and Medicare and Medicaid enrolled populations in the US.

**Methods:**

We retrospectively examined medical claims with an IIM diagnosis (ICD-9-CM 710.3 [dermatomyositis (DM)], 710.4 [polymyositis (PM)], 728.81[interstitial myositis]) in the MarketScan® databases to identify age- and gender-adjusted annual IIM incidence and prevalence for 2004–2008. Sensitivity analysis was performed for evidence of a specialist visit (rheumatologist/ neurologist/dermatologist), systemic corticosteroid or immunosuppressant use, or muscle biopsy.

**Results:**

We identified 2,990 incident patients between 2004 and 2008 (67% female, 17% Medicaid enrollees, 27% aged ≥65 years). Overall adjusted IIM incidence for 2004–2008 for commercial and Medicare supplemental groups combined were 4.27 cases (95% CI, 4.09-4.44) and for Medicaid, 5.23 (95% CI 4.74-5.72) per 100,000 person-years (py). Disease sub-type incidence rates per 100,000-py were 1.52 (95% CI 1.42-1.63) and 1.70 (1.42-1.97) for DM, 2.46 (2.33-2.59) and 3.53 (3.13-3.94) for PM, and 0.73 (0.66-0.81) and 0.78 (0.58-0.97) for interstitial myositis for the commercial/Medicare and Medicaid cohorts respectively. Annual incidence fluctuated over time with the base MarketScan populations. There were 7,155 prevalent patients, with annual prevalence ranging from 20.62 to 25.32 per 100,000 for commercial/Medicare (83% of prevalent cases) and from 15.35 to 32.74 for Medicaid.

**Conclusions:**

We found higher IIM incidence than historically reported. Employer turnover, miscoding and misdiagnosing, care seeking behavior, and fluctuations in database membership over time can influence the results. Further studies are needed to confirm the incidence and prevalence of IIM.

## Background

Autoimmune idiopathic inflammatory myopathies (IIMs) consist of a rare group of autoimmune syndromes characterized by chronic muscle inflammation and muscle weakness. They are classified as autoimmune disorders [1] and have no known cause, although research has implicated infectious agents, medications, and ultraviolet radiation in their etiology [2]*.* Three main types of IIMs have been proposed based on clinical and histopathologic features: (a) polymyositis (PM), (b) dermatomyositis (DM), and (c) sporadic inclusion body myositis (sIBM). Recent reports have described two new types: nonspecific myositis, and immune-mediated necrotizing myopathy [3-5]*.* Older reports [6] also describe an “interstitial myositis” that has not been reported extensively in the literature. Dermatomyositis is the most common of the myositis disorders [2,7]*.* Polymyositis is often associated with other connective tissue disorders such as lupus [6,8,9]*.* Treatments for IIMs typically consist of medications such as corticosteroids as first line therapy, (e.g., high doses of oral or intravenous prednisone) and immunosuppressants and immunomodulators (e.g., methotrexate, mycophenolate mofetil, tacrolimus, rituximab) as second line [2]*.* Physical therapy is also used [9]*.*

There are few epidemiological studies on IIMs. Until recently, many of the studies of incidence and prevalence of the disease have been on relatively small population studies, often using both medical records and muscle biopsy to ascertain the disease [8]*.* These types of studies have identified annual myositis (including its sub-types) incidence in the range of 0.1 to 1 per 100,000 person-years (py) [2,10-15]*.*

One recent study using administrative claims data for Quebec (approximately 7.5 million beneficiaries) estimated prevalence of 21.5 (95% CI: 19.4, 23.9) per 100,000 patients for dermatomyositis and polymyositis, using an IIM case definition of ≥2 outpatient claims with an ICD-9-CM diagnosis recorded for either condition ≤2 months apart and within a 2 year time span, or ≥1 inpatient claim with either ICD-9-CM code, as well as an alternate algorithm with one or more billing code from a rheumatologist [16]*.* The authors reported higher prevalence for women and older patients. Other studies reporting rates by age and/or sex also have found that incidence rates increase with age (although dermatomyositis affects children as well as adults) and are approximately twice as high among women as among men [2,6-8,12]*.* Separate community-based epidemiologic studies identified a range of prevalence for IIMs, including IIM prevalence of 5.1 per 100,000 in the U.S. [17]*,* PM prevalence of 3.45 per 100,000 in Olmsted County, Minnesota, USA [10] and DM prevalence of 21.42 [18]*.*

Recent work using an U.S. privately insured managed care administrative claims database for 2003–2008 identified a 65/35 female to male ratio of patients with myositis (polymyositis, dermatomyositis, or interstitial myositis) with a median age of 49 years. The overall age- and sex-adjusted myositis incidence rate was 6.57 cases (95% CI, 6.20–6.94) per 100,000 py and was 3.79 for polymyositis, 1.38 for dermatomyositis, and 1.69 for interstitial myositis per 100,000 py[19]*,* notably higher than the 0.1 to 0.5 per 100,000 myositis incidence reported using smaller, fixed populations [13-15]. Annual myositis prevalence identified in the same study for the period 2003–2008 ranged from 13.99 in 2003 to 17.37 in 2008 per 100,000 py[19]*.* These prevalence rates are in the range of those reported by Bernatsky [16] in an administrative claims database in Quebec, suggesting prevalence of myositis, as identified in administrative claims databases, may be in the range of 14–22 cases per 100,000 py.

The aim of the present study is to examine age and sex adjusted incidence and prevalence of IIM using commercial, Medicare and Medicaid populations. This approach offers several advantages. First, it uses a large, geographically diverse U.S. administrative claims database. Since IIMs are rare, the large sample available in the database allows more robust estimates of incidence and prevalence than in many previous studies, which examined IIM incidence and/or prevalence in smaller populations. Second, this approach provides incidence and prevalence estimates by sex and age group, and includes populations with diverse payers (both commercial and Centers for Medicare & Medicaid Services [CMS]). Third, this study includes outpatient as well as inpatient claims, which limits bias in case identification by providing greater sensitivity in incidence and prevalence estimates than would be possible if limited to hospitalization data only. Many of the published incidence and prevalence studies of IIMs that utilized only hospital data [8] may have under-estimated incidence and prevalence, since myositis often does not require hospitalization.

## Methods

### Study design and subject identification

Patients were selected for the study period 2004–2008 from large administrative claims databases (the *MarketScan® Commercial Claims and Encounters Database, Medicare Supplemental and Coordination of Benefit Database*, and *Medicaid Multi-State Database*), geographically representative of the U.S. population. The commercial database contains information for de-identified, standardized medical and outpatient pharmacy claims data for the population under age 65 covered by private-sector health plans (approximately 14 million enrollees annually). Enrollees are covered under 130 unique carriers representing a variety of plan types, including fee-for-service and capitated health plans. The Medicare database contains healthcare experience for 1.6 million individuals (retired or working-aged) with Medicare supplemental insurance paid for by employers. The Medicaid database contains the pooled healthcare experience of approximately 28 million Medicaid enrollees representing 10 different states. Commercial and Medicare enrollees were reported together in the same tables, with incidence and prevalence reported for the combined groups, as their data was contributed by the same group of employers. Incidence and prevalence for Medicaid enrollees were reported separately.

The *MarketScan® Databases* used in this study are de-identified and fully compliant with the Health Insurance Portability and Accountability Act of 1996. Because this study did not involve the collection, use, or transmittal of individually identifiable data, Institutional Review Board review or approval was not required.

Three types of IIMs were identified for dermatomyositis (DM: ICD-9-CM 710.3), polymyositis (PM: ICD-9-CM 710.4), and interstitial myositis (ICD-6-CM 728.81), using a combination of outpatient and inpatient medical claims (referred to as “diagnostic claims method”), since patients with the condition may not require inpatient treatment [16]*.* While this method decreases specificity, the increase in sensitivity provides additional insight into the potential scope of persons seeking treatment for IIMs in the U.S. population. Sensitivity analyses, which required criteria in addition to diagnoses (e.g., a specialist visit, IIM medication, or a muscle biopsy) were also performed on annual prevalence and combined incidence (2004–2008).

The incidence date was the earliest date of service between 1/1/2004 and 12/31/2008 with a qualifying medical claim with one of the three IIM diagnoses. A qualifying IIM medical claim was the earliest of an inpatient claim or the first of at least two office or ER visits (including combinations) at least 30 days and less than 365 days apart. The IIM incident cohort was identified as patients at least 18 years of age on the qualifying date of service with an IIM diagnosis, had continuous health plan medical and pharmacy benefits for 1 year before and 1 year after the service date, and had no claim with an IIM diagnosis in a previous year. Incidence was reported annually and for the combined period 2004–2008. The databases extended to 2003, with patients having any evidence of IIMs excluded from the incidence but not excluded from the prevalence cohort. For sensitivity analyses that required either a one year preview or one year follow-up period after the first IIM service date, patient data were evaluated starting in 2003 and extending through 2009.

IIM prevalence was calculated to represent the actual burden of disease at a point of time, and was identified similarly to incidence, except that patients were permitted to have a previous IIM claim. Patients required a qualifying IIM claim during the year, as well as healthplan eligibility for the entire year, to be counted as prevalent in that year. This method identifies prevalence of healthcare utilization for IIM and therefore under-represents IIM prevalence in the MarketScan databases for those patients who have the condition but do not have a qualifying IIM claim in each year for which they maintain healthcare eligibility. However, the strength of this method is that it enables each year to be evaluated separately, and does not result in an artificial increase in prevalence that might be observed if patients with IIM claims in an earlier year in the study continue to contribute to prevalence counts regardless of whether they have an IIM claim in that year.

### Subject characteristics

Patients with IIMs were classified into a general group (any myositis) as well as at least one sub-group: DM, PM, or interstitial myositis. Patients could have more than one type of myositis.

Patient age, gender, urban/rural and geographic region (not available for Medicaid), healthplan type, race (Medicaid only) and payer (commercial, Medicare, Medicaid) were identified on the date of the earliest qualifying IIM claim for both the incident and prevalent cohorts.

Patient comorbidities were identified based on the presence of one inpatient or two or more outpatient claims with a non-rule out ICD-9-CM diagnosis for the comorbidity of interest in the 1 year before the incidence or prevalence date. Medications of interest were identified from outpatient pharmacy claims on or 1 year following the incidence or prevalence date. For the sensitivity analyses, evidence of an outpatient visit to a rheumatologist, dermatologist, or neurologist was captured from medical claims on and 1 year following the index date. Two additional sensitivity criteria were examined: evidence of a medical or outpatient pharmacy claim for a systemic corticosteroid or immunosuppressant (IIM treatment)^a^ on or 1 year following the index date, as well as a medical claim for muscle biopsy^b^ 1 year before, on, or 1 year following the index date. Sensitivity analyses were performed on the combined myositis group as well as on the DM, PM, and interstitial myositis sub-groups.

### Incidence and prevalence calculations

For each calendar year between 2004 and 2008, annual incidence was calculated by dividing the number of incident cases by the corresponding annual population at risk. Prevalent patients were excluded from the numerator as well as the denominator for all incidence calculations. To qualify for the denominators, patients had to have no previous qualifying IIM claims, be 18 years during the calendar year of interest, and have continuous medical and pharmacy eligibility for two consecutive years, including the entire calendar year of interest as well as one day in each of the bordering years.

Incidence was also calculated for the period 2004–2008, using the number of unique incident cases during the time period as numerator. The denominator for the overall incidence rate was the total number of person-years at risk between 2004–2008, for a maximum possible of five person-years for continuous enrollees 18 years or older and with no evidence of an IIM during the period.

For comparison among different data sources, both overall and annual incidence rates were age- and sex-adjusted to a U.S. standard population (2000 U.S. Census using the following formula following Insigna et al. [20]*:*

(1) $$\frac{{\text{n}}_{\text{ij}}\left(\text{U.S}\right)}{\text{N}\left(\text{U.S}\right)}\times \frac{\text{N}\left(\text{MarketScan}\right)}{{\text{n}}_{\text{ij}}\left(\text{MarketScan}\right)}$$

where N is the total population in person-time (in the relevant *MarketScan Database* or in the 2000 U.S. Census as indicated) and nij is the person-time in the specific age and sex strata (from the relevant *MarketScan Database* or the 2000 U.S. Census as indicated).

Annual prevalence was calculated by dividing the number of prevalent cases identified in the data each year by the number of enrollees 18 years or older during the calendar year who had two years of continuous medical and pharmacy eligibility, one of which spanned the entire calendar year of interest, as well as one day in each of the bordering years.

The same methods were applied for sensitivity analyses for the combined 2004–2008 incidence rates and for annual prevalence. Each of the three sensitivity criteria were applied separately as well as combined (patients must have had at least one of the three criteria in addition to being identified as incident or prevalent to contribute to the numerator). For the purposes of this manuscript, each of the three sensitivity analyses as well as the three combined are presented for 2004–2008 incidence for IIMs and the three sub-types. For annual prevalence, only the combined sensitivity analyses are presented, for combined IIMs only, for which to be counted in numerator, patients must have had at least one claim for a muscle biopsy, specialist visit, or immunosuppressant or systemic corticosteroid in the time periods described above, in addition to being identified as prevalent in that year.

### Statistical analyses

Confidence intervals for the crude rates and age- and gender-adjusted rates were calculated using the normal approximation to the binomial distribution. Uncertainty in each yearly age- and gender- adjusted rate was calculated using basic properties of variance, treating each yearly age- and gender-adjusted rate as a simple linear combination of the crude age- and gender-adjusted rates for that same year. Incidence rates were analyzed with the GENMOD procedure in SAS 9.2 using a negative binomial error distribution, log link function, and log of the corresponding denominator as an offset. Equality of crude incidence rates across age groups and gender was tested using likelihood ratio tests (LRT). Sensitivity results were calculated as described above but with the numerators limited to the appropriate subgroup of subjects.

## Results

### Incident cohort

Among nearly 50,750,000 py in the commercial/Medicare databases and close to 9,860,000 py in the Medicaid database, a total of 2,990 incident cases were identified, 2,477 (83%) in the commercially insured/Medicare supplemental databases and 513 (17%) in the Medicaid database (Table1). Among the incident cases in the combined databases, 1,072 had DM, 1,784 had PM and 476 had interstitial myositis (patients may have had more than one type of myositis). Females comprised 67%, with the largest share of patients in the 55–64 age group (Table1). Among commercially insured or Medicare enrolled females, the mean age was 56.3 years (SD 14.8) and among males, 57.7 years (SD 14.3). Among Medicaid beneficiaries, the mean age was 51.6 years (SD 17.0) and 52.2 years (SD 16.4) among females and males respectively, reflecting the younger age distribution with the Medicaid database.

**Table 1:** Characteristics of myositis incident patients (2004–2008)

	**Commercial and Medicare patients**		**Medicaid patients**	
	**n**	**%**	**n**	**%**
**Myositis (Overall)**^**1**^	2,477	100%	513	100.0%
Dermatomyositis	902	36.4%	170	33.1%
Polymyositis	1,438	58.1%	346	67.4%
Interstitial myositis	401	16.2%	75	14.6%
**Year**				
2004	436	17.6%	190	37.0%
2005	522	21.1%	108	21.1%
2006	502	20.3%	64	12.5%
2007	555	22.4%	87	17.0%
2008	462	18.6%	64	12.5%
**Age**				
18-24	60	2.4%	31	6.0%
25-34	123	5.0%	53	10.3%
35-44	292	11.8%	91	17.7%
45-54	558	22.5%	111	21.6%
55-64	748	30.2%	116	22.6%
65-74	392	15.8	63	12.3
75+	304	12.3	48	9.4%
**Gender**				
Female	1,612	65.1	397	77.4%
Male	865	34.9%	116	22.6%
**Race/Ethnicity (N, %)**				
Caucasian	….	….	193	37.6%
African American	….	….	220	42.9%
Hispanic	….	….	25	4.9%
Other/Unknown	….	….	7	1.4%
**Region**				
Northeast	289	11.7%	….	….
North Central	756	30.5%	….	….
South	956	38.6%	….	….
West	461	18.6%	….	….
Unknown	15	0.6%	….	….
**Urbanization**				
Urban	2,087	84.7%	….	….
Rural	380	15.3%	….	….
Unknown	10	0.4%	….	….
**Medicare**	699	28.2 %	228	44.4%
**Fee-for-service (N, %)**				
Fee-for-service	1,443	58.3%	362	70.6%
Not fee-for-service	1,034	41.7%	150	29.2%
Unknown	0	0%	1	0.2%
**Clinical Conditions (365 days before index date)**				
Rheumatoid Arthritis	170	6.9%	51	9.9%
Systemic lupus erythematosus	158	6.4%	41	8.0%
Systemic sclerosis	44	1.8%	12	2.3%
Cushing syndrome	3	0.1%	1	0.2%
Addison's disease	10	0.4%	6	1.2%
Other Inflammatory Arthritis/Arthropathies	563	22.7%	151	29.4%
Back problems	654	26.4%	139	27.1%
Dysphagia	130	5.2%	29	5.7%
Hypertension	897	36.2%	256	49.9%
Diabetes	385	15.5 %	159	31.0%
Depression	164	6.6%	103	20.1%
Malignancy	259	10.5%	52	10.1%
**Medications on or 365 days after index date**				
Systemic corticosteroid	1852	74.8%	321	62.6%
Topical corticosteroid	1722	69.5%	294	57.3%
Methotrexate	650	26.2%	73	14.2%
Azathioprine	352	14.2%	64	12.5%
Hydroxychloroquine	353	14.3%	51	9.9%
Intravenous immunoglobulin	24	1.0%	6	1.2%
Cyclosporine	62	2.5%	6	1.2%
Mycophenolate mofetil	136	5.5%	16	3.1%
Leflunomide	17	0.7%	5	1.0%
Thalidomide	1	0.0%	0	0.0%
Cyclophosphamide	34	1.4%	9	1.8%
Tacrolimus	50	2.0 %	5	1.0%
Rituximab	24	1.0%	2	0.4%

^1^Myositis subgroups were not mutually exclusive, and patients could be assigned to more than one subgroup.

Among Medicaid beneficiaries, those with incident IIMs were more likely to be African American (42.9%) than Caucasian (37.6%) or Hispanic (4.9%) (Table1). The race/ethnicity composition of the denominator, however, was 22.8%, 41.8%, and 19.7% respectively, indicating that incidence of IIMs was disproportionately higher among African Americans. Race was not available for the commercial/Medicare cohort. The southern region contributed the greatest share of incident cases to the commercial/Medicare cohort, followed by the north central region, with nearly 85% of cases from an urban area, but these differences reflect the overall distribution of patients in the databases.

The most common comorbid conditions (identified in 15-36% of incident patients) among the commercial/Medicare cohort were hypertension, back problems, other inflammatory arthritis/arthropathies (IA/A), and diabetes. Rates of all reported comorbid conditions were higher among the Medicaid cohort, particularly hypertension (49.9%) and diabetes (31%), and followed by IA/A (29.4%). Conversely, medication usage was lower for nearly all drugs among the Medicaid than the commercial/Medicare cohort. Approximately two-thirds or more of all patients received a systemic corticosteroid, followed by a topical corticosteroid. Methotrexate was received among 26.2% of commercial/Medicare incident patients and among 14.2% of Medicaid patients. Azathioprine and hydroxychloroquine were the next most frequently received, with 14.2% and 12.5% receiving azathioprine and 14.3% and 9.9% receiving hydroxychloroquine among the commercial/Medicare and Medicaid populations respectively. Among both cohorts, 2.5% or fewer used intravenous immunoglobulin, cyclosporine, leflunomide, thalidomide, cyclophosphamide, tacrolimus, or rituximab (Table1).

### Incidence rates

As shown in Table2, overall IIM incidence rates for the period 2004–2008 for commercial and Medicare supplemental groups combined were 4.88 (crude) and 4.27 (adjusted) cases (95% CI, 4.09-4.44) and for Medicaid, 5.2 (crude) and 5.23 (adjusted) cases (95% CI 4.74-5.72) per 100,000 py. Adjusted incidence rates per 100,000 py for the same period for each IIM sub-type are as follows: 1.52 (95% CI 1.42-1.63) and 1.70 (1.42-1.97) for DM, 2.46 (2.33-2.59) and 3.53 (3.13-3.94) for PM, and 0.73 (0.66-0.81) and 0.78 (0.58-0.97) for interstitial myositis for the commercial/Medicare and Medicaid cohorts respectively.

**Table 2:** Myositis incidence (2004–2008)

	**Commercial and Medicare supplemental database incidence (per 100,000)**^**1**^			**Medicaid database incidence (per 100,000)**^**1**^		
	**Crude rate**	**Age- and Gender- adjusted rate**^**4**^	**95% CI (adjusted rate)**	**Crude rate**	**Age- and Gender- adjusted rate**^**4**^	**95% CI (adjusted rate)**
**Myositis (Overall)**^**2**^	4.88	4.27	4.09-4.44	5.20	5.23	4.74-5.72
Dermatomyositis	1.78	1.52	1.42-1.63	1.72	1.70	1.42-1.97
Polymyositis	2.83	2.46	2.33-2.59	3.51	3.53	3.13-3.94
Interstitial myositis	0.79	0.73	0.66-0.81	0.76	0.78	0.58-0.97
**Year**						
2004	5.37	4.52	4.07-4.96	4.27	4.47	3.80-5.15
2005	6.02	5.08	4.61-5.54	6.16	6.44	5.08-7.80
2006	5.15	4.45	4.04-4.86	5.33	5.14	3.73-6.54
2007	4.80	4.25	3.87-4.62	7.27	6.85	5.29-8.41
2008	3.66	3.38	3.06-3.71	5.11	5.08	3.70-6.45
**Age**	(LRT χ^2^ =81.73, df = 6 , p < 0.001)^3^			(LRT χ^2^ =38.69, df = 6 , p < 0.001)^3^		
18-24	1.35	1.33	0.99-1.67	2.02	1.74	1.09-2.40
25-34	2.03	1.96	1.61-2.30	3.34	3.62	2.48-4.76
35-44	3.14	3.10	2.74-3.45	6.13	5.33	4.20-6.47
45-54	4.77	4.69	4.30-5.08	7.97	7.54	6.12-8.95
55-64	6.95	6.94	6.44-7.44	10.20	9.72	7.93-11.52
65-74	9.25	9.30	8.38-10.22	5.13	4.83	3.62-6.05
75+	7.21	7.32	6.49-8.15	3.20	3.08	2.18-3.99
**Gender**	(LRT χ^2^ =6.77, df = 1, p =0.009)^3^			(LRT χ^2^ =9.08, df = 1, p =0.003)^3^		
Female	5.97	5.38	5.11-5.66	5.95	6.68	6.00-7.36
Male	3.65	3.07	2.85-3.28	3.64	3.67	2.96-4.37

^1^Rates are per 100,000 person-years for the period from 2004–2008, except year-specific rates, which are per 100,000 individuals.

^2^Myositis subgroups were not mutually exclusive, and patients could be assigned to more than one subgroup.

^3^Generalized linear model using negative binomial family and log link. Not adjusted for other covariates. Year-specific analysis on adjusted rates; demographic analyses on crude rates.

^4^Age and gender adjustment was done for all variables, except age distribution was gender adjusted and gender distribution was age adjusted. Rates reflect 2000 US population.

Adjusted annual incidence for overall IIM ranged from 3.38 (95% CI 3.06-3.71) in 2008 to 5.08 (95% CI 4.61-5.54) in 2005 in the commercial/Medicare database and from 4.47 (95% CI 3.80-5.15) in 2004 to 6.58 (95% CI 5.29-8.41) in 2007 in the Medicaid database. Due to large fluctuations in the size and composition of the denominator associated with variations in MarketScan subscribers (particularly in the Medicaid database), comparisons over time are not meaningful, and no trend analysis was undertaken. Crude and adjusted IIM incidence rates were higher among females than males in both databases. Among commercial/Medicare enrollees, adjusted incidence among females was 5.38 (5.11-5.66) and among males was 3.07 (2.85-3.28) per 100,000 py; LRT χ^2^ =6.77, df = 1, p =0.009 as shown in Table2. Among Medicaid enrollees, rates were 6.68 (6.00-7.36) for females and 3.67 (2.96-4.37) for males LRT χ^2^ =9.08, df = 1, p =0.003 (Table2). IIM incidence rates by age group varied between the two cohorts. In the commercial/Medicare database, the 65–74 years cohort had the highest rates (9.30 per 100,000 py; 95% CI 8.38-10.22), LRT χ^2^ =81.73, df = 6 , p < 0.001, while in the Medicaid database, the 55–64 cohort had the highest rates (9.72 per 100,000 py; 95% CI 7.93-11.52), LRT χ^2^ =38.69, df = 6 , p < 0.001 (Table2).

Sensitivity analyses, which were performed on the 2004–2008 incidence rates for each IIM sub-type, revealed highly variable incidence rates depending on the treatment characteristic used as a sensitivity measure. After the combined-metric sensitivity analysis, which required a qualifying incident myositis claim in addition to either a muscle biopsy in the one year prior to, on, or following the incidence date; or on the incidence date or one year following it, an office visit to a rheumatologist, dermatologist or neurologist; or a medical or pharmacy claim for an immunosuppressant or systemic corticosteroid, reported incidence decreased for all groups. The sensitivity analysis adjusted incidence rates for overall IIMs were 3.29 cases (vs. 4.27) per 100,000 py for commercial/Medicare and 3.5 cases (vs. 5.23) per 100,000 py for Medicaid (Table3). In other words, after applying the combined sensitivity criteria, the number of identified incident cases per 100,000 py decreased by 23% for commercial/Medicare and by 33% for Medicaid.

**Table 3:** Sensitivity Analysis of Incidence Rates per 100,000 person-years (2004–2008)

	**Commercial and Medicare supplemental database**				**Medicaid database**			
	**N**	**Crude rate**	**Age & Gender adjusted rate**	**95% CI for adjusted rate**	**N**	**Crude rate**	**Age & Gender adjusted rate**	**95% CI for adjusted rate**
**Base case analysis (IIMs)**	2,477	4.88	4.27	4.09-4.44	513	5.20	5.23	4.74-5.72
Dermatomyositis	902	1.78	1.52	1.42-1.63	170	1.72	1.70	1.42-1.97
Polymyositis	1,438	2.83	2.46	2.33-2.59	346	3.51	3.53	3.13-3.94
Interstitial myositis	401	0.79	0.73	0.66-0.81	75	0.76	0.78	0.58-0.97
**Presence of muscle biopsy one year prior to or one year following index date**								
IIMs	738	1.45	1.25	1.16-1.35	102	1.03	1.05	0.83-1.27
Dermatomyositis	248	0.49	0.43	0.38-0.49	34	0.34	0.34	0.22-0.46
Polymyositis	574	1.13	0.96	0.88-1.04	88	0.89	0.91	0.70-1.11
Interstitial myositis	21	0.04	0.04	0.02-0.06	2	0.02	0.02	0.01-0.04
**Presence of specialist visit on index date or in the year following**								
IIMs	535	1.05	0.91	0.82-0.99	64	0.65	0.63	0.46-0.79
Dermatomyositis	207	0.41	0.35	0.30-0.40	21	0.21	0.24	0.13-0.35
Polymyositis	374	0.74	0.63	0.56-0.70	49	0.50	0.46	0.32-0.59
Interstitial myositis	27	0.05	0.05	0.03-0.07	8	0.08	0.06	0.02-0.10
**Presence of immunosuppressants and/or systemic corticosteroids one year following index date**								
IIMs	1,761	3.47	3.01	2.86-3.16	304	3.08	3.07	2.70-3.44
Dermatomyositis	725	1.43	1.24	1.14-1.33	103	1.04	1.02	0.80-1.23
Polymyositis	1,146	2.26	1.96	1.84-2.08	225	2.28	2.29	1.97-2.61
Interstitial myositis	133	0.26	0.23	0.19-0.27	32	0.32	0.31	0.20-0.43
**Presence of any of the above (combined sensitivity criteria)**								
IIMs	1,917	3.78	3.29	3.13-3.44	347	3.52	3.50	3.10-3.89
Dermatomyositis	758	1.49	1.29	1.20-1.39	115	1.17	1.13	0.91-1.36
Polymyositis	1,250	2.46	2.14	2.01-2.26	257	2.61	2.62	2.28-2.97
Interstitial myositis	160	0.32	0.28	0.24-0.33	38	0.38	0.36	0.24-0.48

The combined sensitivity analysis identified between 85% and 87% of incident DM and PM commercial/Medicare patients as compared to the diagnostic claims method, and only 38% of those with incident interstitial myositis. In the Medicaid population the sensitivity analysis identified 66% of incident DM patients, 74% of incident PM patients, and 46% of incident interstitial myositis patients reported as incident using the diagnostic claims method. Among the individual sensitivity criteria, specialist visits on or one year after the incidence date identified the lowest percentage of incident patients and immunosuppressants or systemic corticosteroids on or one year after identified the largest percentage (Table3).

### Prevalent cohort

We identified 7,155 prevalent patients over the period 2004–2008. Among these patients, 39.1% had DM, 58.9% had PM, and 9.8% had interstitial myositis, with some patients being prevalent for more than one type of IIM (Table4). In the commercial/Medicare database, annual counts of patients identified as prevalent for interstitial myositis were low, ranging from 102 in 2004 to 184 in 2006. In the Medicaid database, annual prevalence counts for DM and interstitial myositis were also low. Prevalent DM cases ranged from 246 in 2004 (when the Medicaid database was the largest) to 109 in 2008, and prevalent interstitial myositis cases ranged from a high of 62 in 2004 to only 13 cases in 2008. Therefore the annual prevalence results focus on combined IIMs rather than the individual sub-types.

**Table 4:** Characteristics of Myositis Prevalent Patients (2004–2008)

	**Commercial and Medicare patients**		**Medicaid patients**	
	**n**	**%**	**n**	**%**
**Myositis (Overall)**^**1**^	5,941	100.0%	1,214	100.0%
Dermatomyositis	2,361	39.7%	440	36.2%
Polymyositis	3,443	58.0	771	63.5
Interstitial myositis	585	9.8%	115	9.5%
**Year**^**2**^				
2004	1,865	31.4%	684	56.3%
2005	2,197	37.0%	574	47.3%
2006	2,242	37.7%	355	29.2%
2007	2,562	43.1%	335	27.6%
2008	2,605	43.8%	306	25.2%
**Age**				
18-24	249	4.2%	104	8.6%
25-34	334	5.6%	145	11.9%
35-44	774	13.0%	254	20.9%
45-54	1,403	23.6%	264	21.7%
55-64	1,773	29.8%	248	20.4%
65-74	810	13.6%	118	9.7%
75+	598	10.1%	81	6.7%
**Gender**				
Female	4,029	67.8%	928	76.4%
Male	1,912	32.2%	286	23.6%
**Race/Ethnicity (N, %)**				
Caucasian	….	….	440	36.2%
African American	….	….	540	44.5%
Hispanic	….	….	81	6.7%
Other/Unknown	….	….	15	1.2%
**Region**				
Northeast	652	11.0%	….	….
North Central	1,668	28.1%	….	….
South	2,416	40.7%	….	….
West	1,167	19.6%	….	….
Unknown	38	0.6 %	….	….
**Urbanization**				
Urban	4,991	84.0%	….	….
Rural	917	15.4%	….	….
Unknown	33	0.6%	….	….
**Medicare**	1,417	23.9%	460	37.9 %
**Fee-for-service (N, %)**				
Fee-for-service	3,639	61.3 %	850	70.0%
Not fee-for-service	2,302	38.7%	363	29.9%
Unknown	0	0%	1	0.1%
**Clinical Conditions**				
Rheumatoid Arthritis	349	5.9%	114	9.4%
Systemic lupus erythematosus	317	5.3%	111	9.1%
Systemic sclerosis	93	1.6%	34	2.8%
Cushing syndrome	6	0.1%	7	0.6%
Addison's disease	16	0.3%	7	0.6%
Other Inflammatory Arthritis/Arthropathies	983	16.5%	309	25.5%
Back problems	1074	18.1%	249	20.5%
Dysphagia	213	3.6%	67	5.5%
Hypertension	1578	26.6%	494	40.7%
Diabetes	764	12.9%	299	24.6%
Depression	299	5.0%	191	15.7%
Malignancy	467	7.9%	94	7.7%
**Medications on or 365 days after index date**				
Systemic corticosteriods	4,507	75.9%	881	72.6%
Topical corticosteroids	4,155	69.9%	841	69.3%
Methotrexate	1,887	31.8%	281	23.1%
Azathioprine	993	16.7%	234	19.3%
Hydroxychloroquine	926	15.6%	142	11.7%
Intravenous immunoglobulin	79	1.3%	20	1.6%
Cyclosporine	202	3.4%	17	1.4%
Mycophenolate mofetil	402	6.8%	61	5.0%
Leflunomide	73	1.2%	15	1.2%
Thalidomide	4	0.1 %	0	0.0 %
Cyclophosphamide	83	1.4%	25	2.1%
Tacrolimus	158	2.7%	11	0.9%
Rituximab	48	0.8%	4	0.3%

^1^Myositis subgroups were not mutually exclusive, and patients could be assigned to more than one subgroup.

^2^Yearly counts reflect number of prevalent patients identified in each year and are not mutually exclusive.

Prevalent cases were distributed relatively evenly by year among the commercial/Medicare database, but in the Medicaid database were highest in 2004 followed by 2005, which reflected the notably higher base population in those years rather than any observed trend in prevalence over time. As with incidence, more than 2/3 of the prevalent population were females (68% among the commercial/Medicare and 76% among the Medicaid databases). The mean age of prevalent patients was slightly lower than that of incident patients. Among commercially insured or Medicare enrolled females, the mean age was 54.5 years (SD 15.2) and among males, 55.4 years (SD 14.7). Among Medicaid beneficiaries (a younger group overall), the mean age for females was 49 years (SD 16.4) and for males was 47.7 years (SD 16.3). As with incidence, Medicaid enrollees with prevalent IIMs were disproportionately African American (44.5%) as compared to Caucasian (36.2%) or Hispanic (6.7%). The regional and urban/rural distribution of prevalent cases was similar to incident cases in the commercial/Medicare population, with a slightly higher percentage in the southern and western regions in the prevalent cohort than in the incident cohort (Table4). These distributions reflected the source databases.

Comorbid conditions identified in the prevalent population were similar in proportion to those identified in the incident population with hypertension, back problems, other inflammatory arthritis/arthropathies, and diabetes the most common conditions among both the commercial/Medicare and Medicaid cohorts. Medication usage for nearly all reported drugs was slightly higher among the prevalent than the incident populations for both databases (Table4).

Annual prevalence over the study period of 2004–2008 ranged from 20.62 to 25.32 per 100,000 for commercial/Medicare enrollees (83% of prevalent cases) and from 15.35 to 32.74 for Medicaid enrollees (Table5). After sensitivity analysis, the number of reported annual prevalent patients decreased to 18.47 in 2004 and 21.63 in 2008 for the commercial/Medicare cohort, a difference of 10% to 15%. For the Medicaid cohort, after applying the combined sensitivity criteria, annual prevalence dropped to 12.39 in 2004 to 28.75 in 2005, reflecting declines in prevalence of 12% to 22%.

**Table 5:** Annual myositis prevalence (2004–2008): Base case and sensitivity analysis

**Year**	**Commercial and Medicare supplemental database (per 100,000 individuals)**	**Annual prevalence 95% CI**	**Medicaid database (per 100,000 individuals)**	**Annual prevalence 95% CI**
**Base case analysis**				
2004	22.96	21.92-24.01	15.35	14.20-16.50
2005	25.32	24.26-26.38	32.74	30.06-35.42
2006	22.99	22.04-23.94	29.56	26.49-32.64
2007	22.15	21.29-23.00	27.98	24.99-30.98
2008	20.62	19.83-21.41	24.42	21.68-27.15
**Sensitivity analysis**^**1**^				
2004	19.43	18.47-20.39	12.39	11.36-13.42
2005	21.63	20.66-22.61	28.75	26.24-31.25
2006	19.71	18.82-20.59	23.98	21.21-26.75
2007	19.46	18.65-20.26	22.55	19.86-25.24
2008	18.47	17.72-19.22	19.15	16.73-21.57

^1^Myositis patients with presence of muscle biopsy on or within one year before or after prevalence date; specialist visit on or within one year after the prevalence date; presence of immunosuppressants and/or systemic corticosteroids on or within one year after index date. Specialist included rheumatologist, dermatologist, or neurologist.

**Figure 1:**
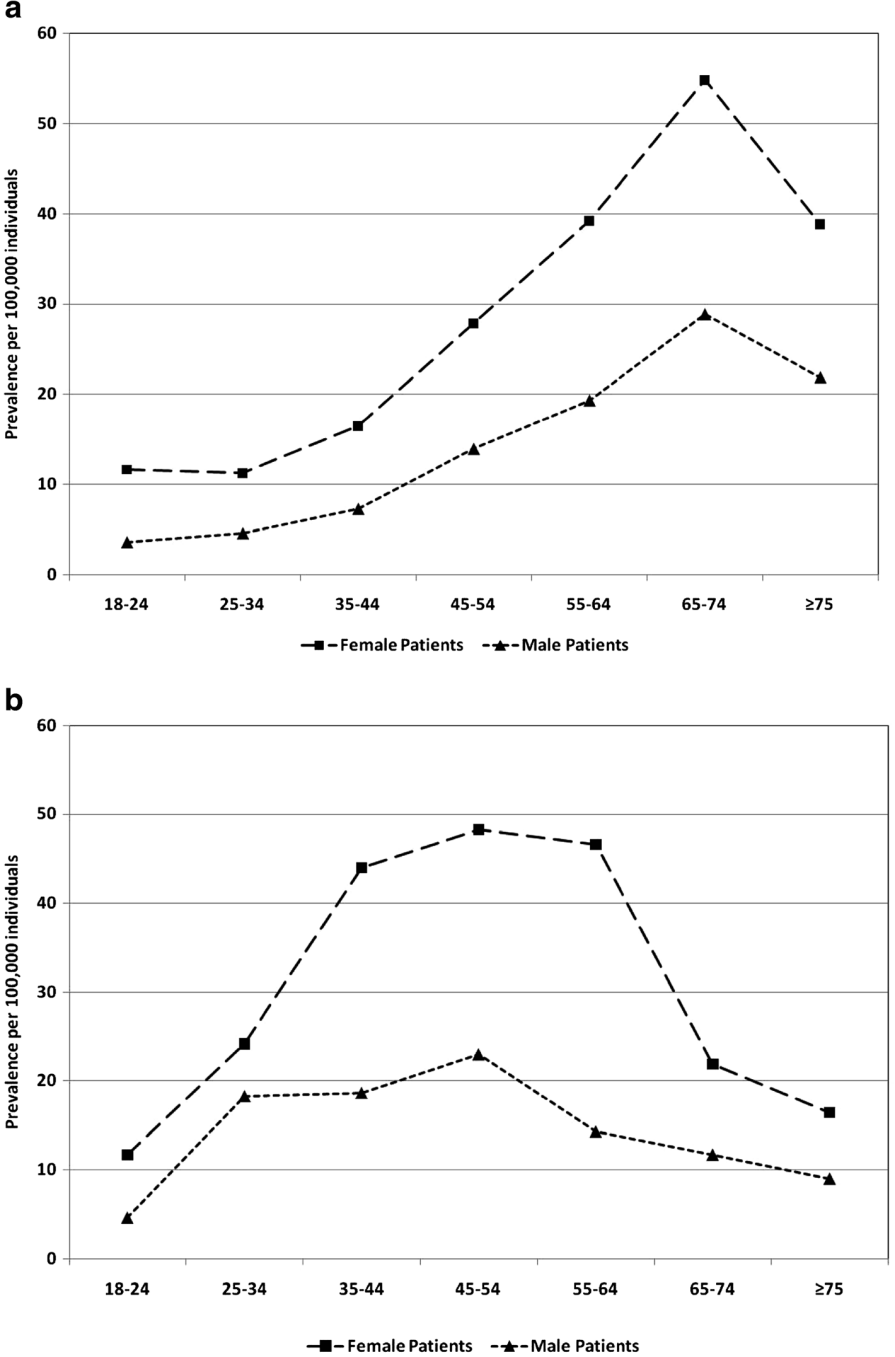
a. 2008 myositis prevalence by age: commercial and medicare databases. b. 2008 myositis prevalence by age: medicaid database.

Prevalence was higher among females than males in all years and for both commercial/Medicare and Medicaid databases. For example, in 2008, the most recent year for which prevalence data were available, female prevalence per 100,000 by age group varied from 11.23 among those 25–34 years of age to 54.87 among those 65–74 years of age, while for males prevalence ranged from 3.58 among 18–24 year olds to 28.83 among 65–74 year olds in the commercial/Medicare population (Figure1a). Similarly, among the Medicaid population, prevalence in 2008 ranged from 11.7 in the 18–24 years cohort to 48.27 in the 45–54 years cohort among females and from 4.61 to 22.99 in the same age groups among males (Figure1b). In 2008, prevalence for DM was 9.18 per 100,000 (13.7 in females, 4.03 in males), for PM was 11.19 (12.2 in females and 8.9 in males), and for interstitial myositis was 1.12 (1.46 in females, 0.74 in males) in the commercial/Medicare population (results not shown). For 2008, prevalence for DM was 8.7 per 100,000 (11.24 in females, 3.26 in males), for PM was 15.48 (18.15 in females and 9.77 in males), and for interstitial myositis was 1.04 (0.82 in females, 1.5 in males) in the Medicaid population (results not shown). Annual observed counts of DM, PM, and in particular, interstitial myositis were rare in the Medicaid population, with in some cases two, one, or zero observations in a given age/gender category. Thus annual DM, PM, and interstitial myositis prevalence in the Medicaid population was subject to instability due to low counts.

## Discussions

IIMs are a rare condition, and little is known about the extent of their incidence and prevalence in the U.S. population. In this study, we used the MarketScan Commercial, Medicare, and Medicaid administrative claims databases to identify incidence and prevalence of IIMs based on medical claims with an ICD-9-CM diagnosis indicating DM, PM, or interstitial myositis. Administrative claims analyses, such as the one performed here, have the benefit of data contributed by very large populations seeking medical care, which can be used to identify rare conditions and shed additional light into incidence and prevalence. The limitation of these methods is the flip side of the benefit coin, in that unless patients seek care for their IIM, they will not be correctly identified as incident or prevalent. In addition, misdiagnosis or recording errors can result in under- or over-reporting of the condition. Another limitation in using administrative claims databases for identifying incidence and prevalence is that the results are sensitive to the methods used to identify the population denominator.

Due to the dynamic nature of the employer-based and Medicaid administrative claims databases used here, the annual values are most informative as snapshots providing a range of estimates among a fluctuating population, rather than indicative of trends in incidence or prevalence of myositis in the U.S. population. Another consideration particular to employer-based databases is that employees with chronic conditions may be less likely to change employers and risk changes to their healthcare coverage, and therefore may stay in the data longer than other employees at the same employer.

Both the current study and one using very similar methods (1 inpatient or 2 outpatient claims for PM, DM, or interstitial myositis) in another U.S. managed care administrative claims database [19] reported notably higher incidence of IIMs than the 0.1 to 1 per 100,000 py reported in community-based studies using smaller populations [2,10-15]*.* This study identified age- and sex-adjusted incidence rates of 4.27 (commercial/Medicare) and 5.23 (Medicaid) per 100,000 py for the period 2004–2008, while Furst et al. [19] reported adjusted incidence rates of 6.57 (95% CI, 6.20–6.94) per 100,000 py for combined IIMs. For DM, we identified adjusted incidence rates of 1.52 and 1.70 per 100,000 py in the commercial/Medicare and Medicaid databases respectively, which were relatively comparable to the 1.38 per 100,000 py reported by Furst et al. [19]*.* Incidence rates for PM were also comparable at 2.46 (commercial/Medicare) and 3.53 (Medicaid) in this study versus 3.79 per 100,000 py in Furst et al [19]*.* These rates were similar to the prevalence of PM of 3.45 per 100,000 py identified by Wilson et al. [10]*.* Interstitial myositis varied among the two studies, in that it was the least common of the three types of IIM, with adjusted incidence of 0.73 and 0.78 per 100,000 py in the two databases compared to 1.69 per 100,000 py reported in Furst et al*.*[19]*,* in which DM rather than interstitial myositis was the least common type of myositis. Due to very low counts of interstitial myositis, along with limited consistent clinical information about the condition and its diagnosis, incidence and prevalence for that sub-type should be interpreted with caution.

In contrast to incidence estimates, the prevalence of IIMs identified by medical claim (20.62 to 25.32 per 100,000 py in commercial/Medicare and 15.35 to 32.74 in Medicaid) in this study is quite comparable to that identified using a contemporary administrative claims database in Quebec (combined DM and PM annual prevalence in 2003 of 21.5 (95% CI 19.4-23.9) per 100,000 py [16]*.* The prevalence of IIMs identified in this study is, however, somewhat higher than prevalence of combined DM, PM and interstitial myositis given in Furst et al. [19] of 9.54 in 2003 and 13.61 in 2008, albeit the lower prevalence in Furst et al. could be associated with a younger population (9% of prevalent cases were ≥ 65 years in their IIM cohort vs. 24% ≥ 65 years in the commercial/Medicare and 16% of prevalent cases in the Medicaid IIM cohorts). Notably, all three administrative claims studies reported annual prevalence that was nearly 2- to 6-fold higher than that reported in one earlier study from 2003 [17]*.* It is possible that with increased awareness of IIMs, the condition may be increasingly recognized and diagnosed, and thus showing up more frequently in administrative claims databases over the past decade than in previous decades.

When the 2004–2008 age- and sex-adjusted incidence rates for the two databases are projected to the U.S. population ages 18 years and older, the expected number of incident cases per year ranges from 8,919 to 10,937 (based on the commercial/Medicare and Medicaid incidence rates respectively, standardized to the 2000 U.S. Census). Using the combined sensitivity analysis (e.g., IIM incidence along with one or more of the three sensitivity criteria), the expected number of incident cases per year among adults in the U.S. ranges from 6,871 (commercial/Medicare incidence rates) to 7,312 (Medicaid incidence rates).

Extrapolating the annual prevalence of persons seeking medical care for IIMs reported here to the U.S. population (as of the 2000 U.S. Census) aged 18 years and older from the commercial/Medicare cohort results in a range of an estimated 43,000 to 53,000 treated cases per year, or, after employing any one of the three sensitivity criteria, an estimated 38,000 to 45,000 treated cases per year. If the Medicaid annual prevalence is used, estimated annual U.S. IIM prevalence ranges from approximately 32,000 to 68,000 using IIM claims only, or 26,000 to 60,000 treated cases using any of the three sensitivity criteria.

This study is unique in its incorporation of three payer groups: commercially insured, Medicare enrollees (with employer paid supplemental insurance), and Medicaid enrollees. The different incidence and prevalence identified in the commercial/Medicare versus Medicaid populations in this analysis may be a result of differences in patients’ healthcare-seeking behaviors as well as in reimbursement and practice patterns rather than the underlying difference in disease incidence and prevalence among the two populations. The relative similarity in prevalence and incidence identified in separate studies conducted in different administrative claims databases indicates that these variations are common in these types of databases. Another consideration unique to the current study is the high incidence and prevalence among African Americans identified in the Medicaid database. Since race/ethnicity is unknown in the commercial and Medicare cohorts, it is unknown to what extent differences in racial composition between the two cohorts may influence the observed incidence and prevalence of myositis. Additional studies of IIM incidence and prevalence by race/ethnicity are needed.

The incidence and prevalence estimates reported here are in the range of those reported in other studies using administrative claims databases. Like those studies, the estimates reported here are based on the presence of a medical claim with the ICD-9-CM diagnoses of interest and are a function of individuals seeking medical care and physician reporting of diagnosis. This study used an algorithm to exclude rule-out diagnoses; nevertheless, administrative claims are not a substitute for a complete medical record for the study population and thus mis-diagnoses are possible. Since a one year “clean” period was required to qualify as incident, incidence may have been over-estimated if patients already had an IIM but did not receive medical care in the preceding year. In addition, prevalence was expected to be under-estimated as it was defined by evidence of a qualifying medical claim with an IIM diagnosis in each year. Patients with the condition who did not seek medical care in a given year would not be captured in the prevalence estimates. Finally, employer turnover, miscoding and misdiagnosing, care seeking behavior, and fluctuations in database membership over time can influence the results. Further studies are needed to confirm the incidence and prevalence of IIM.

## Conclusions

Among 2,990 incident IIM patients between 2004 and 2008, overall adjusted IIM incidence for 2004–2008 for commercial and Medicare supplemental groups combined were 4.27 cases per 100,000 py, and for Medicaid were 5.23 cases per 100,000 py. The highest incidence among disease sub-types was for PM, at 2.46 (commercial/Medicare) and 3.53 (Medicaid) new cases per 100,000 py. Annual IIM prevalence ranged from 20.62 to 25.32 per 100,000 for commercial/Medicare (83% of prevalent cases) and from 15.35 to 32.74 for Medicaid. Although this study found higher IIM incidence than historically reported based on smaller, localized populations, the similarity of the findings of the current study with others in the literature using administrative claims databases suggests that IIM incidence in the U.S. population is in the range of 3 to 7 new cases per 100,000 py and that prevalence as measured by claims for IIM treatment is in the range of 32,000 to 68,000 per year. Future studies using alternate administrative claims databases will be helpful in narrowing down the range of incidence and prevalence estimates, and additional analyses of the cohorts identified as incident and prevalent using chart review would be beneficial in confirming IIM diagnoses, particularly for the three sub-types.

## Endnotes

^a^Immunosuppressants included methotrexate, azathioprine, hydroxychloroquine, intravenous immunoglobulin, cyclosporine, mycophenolate mofetil, leflunomide, thalidomide, cyclophosphamide, tacrolimus, and rituximab.

^b^Muscle biopsy codes included ICD-9-CM Procedure 83.21 or CPT codes 10021, 10022, 20200, 20205, 20206, 21550, 21925, 23066, 24066, 25066, 27041, 27324, or 27614.

## Competing interests

This study was supported by MedImmune LLC. Dr. Amato has received research funding from MedImmune LLC. When this study was performed, Dr. Tomic was an employee of Thomson Reuters, which has received funding from MedImmune LLC. Dr. Fernandes is an employee of MedImmune LLC.

## Authors’ contributions

KEST contributed to the study design, carried out the analysis and interpretation of the administrative claims data, and drafted the manuscript. AWF and AAA conceived of the study and its design and were involved in critical review and revision of the manuscript. All authors read and approved the final manuscript.

## Pre-publication history

The pre-publication history for this paper can be accessed here:

http://www.biomedcentral.com/1471-2474/13/103/prepub
